# Cardiovascular disease and compliance with lipid-lowering therapy among young individuals with familial hypercholesterolemia in Norway – A register study

**DOI:** 10.1016/j.ajpc.2025.101043

**Published:** 2025-06-18

**Authors:** Gisle Langslet, Emil A. Asprusten, Jannicke Igland, Kirsten B. Holven, Martin P. Bogsrud, Kjetil Retterstøl

**Affiliations:** a Lipid Clinic, Oslo University Hospital, Postbox 4959, Nydalen, NO-0424, Oslo, Norway; b Department of Global Public Health and Primary Care, University of Bergen, Postbox 7804, NO-5020, Bergen, Norway; c Department of Health and Caring Sciences, Western Norway University of Applied Sciences, Postbox 7030, 5020, Bergen, Norway; d Norwegian National Advisory Unit on Familial Hypercholesterolemia, Oslo University Hospital, Oslo, Norway; e Department of Nutrition, Institute of Basic Medical Sciences, University of Oslo, Postbox 1046, Blindern, NO-0317, Oslo, Norway; f Unit for Cardiac and Cardiovascular Genetics, Oslo University Hospital, Postbox 4956, Nydalen, NO-0424, Oslo, Norway

**Keywords:** Familial hypercholesterolemia, Lipid lowering therapy, Cardiovascular disease, All-cause death

## Abstract

**Aims:**

Investigation of cardiovascular disease (CVD), all-cause death and use of statins and ezetimibe among young Norwegian individuals with heterozygous Familial hypercholesterolemia (FH).

**Methods:**

We included subjects with genetically verified FH born 1988–2008 and twenty controls per FH subject, with linkage to prescription data, hospitalization data and cause of death from national Norwegian health registries. Data on CHD and death during 2008–2018, and for dispensed prescriptions during 2004–2018, were collected.

**Results:**

1351 subjects with FH and 27,015 controls were included. Mean age (SD) at start of follow-up was 12.3 (5.4) years. There was one Coronary Heart Disease (CHD) event and 6 deaths in the FH-group and 3 CHD events and 53 deaths in the control group. CHD and all-cause death were non-significantly increased in the FH-group, hazard ratios (95 % confidence intervals) 6.68 (0.69–64.20) and 2.26 (0.98–5.28), respectively. None of the deaths in the FH-group were related to cardiovascular disease. 83 % of subjects with FH had been prescribed a statin, and 21 % ezetimibe. During the first year after the first prescription, 18.5 % of subjects did not refill their prescription within 180 days after the end date of the previous prescription. 69 % and 60 % of subjects with a prescription had >80 % of days covered with statins and ezetimibe, respectively. After 8 years, around 70 % of subjects were covered with statins.

**Conclusions:**

Results suggest increased risk of CHD in FH relative to controls, but measures are imprecise because of low absolute risks. Compliance with lipid lowering therapy was moderate.

## Highlights

Investigation of CV disease during 2008-2018, and use of lipid lowering drugs during 2004–2018 among 1351 young subjects with FH.

• There was one cardiovascular event and 6 deaths among subjects with FH, and three CV events and 53 deaths among a general population control group.
• None of the deaths among the FH subjects were due to CV disease.
• Results may suggest increased risk of CV events among subjects with FH, but the number of events was too low to reliably determine this.
• Statins were prescribed to 83 % and ezetimibe to 21 % of subjects, after one year 85 % were covered with statins, decreasing to 70 % after 8 years.

## Graphical abstract

**Figure:**
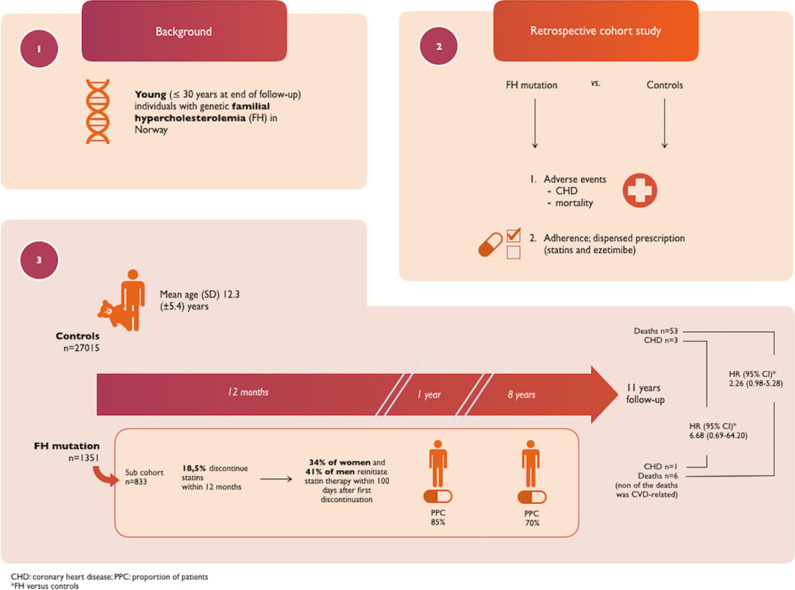

## 1 Introduction

Heterozygous Familial hypercholesterolemia (FH) is a common autosomal dominant condition with an estimated prevalence of 1 in 300 individuals in a variety of populations [1]. Low-density lipoprotein (LDL) cholesterol levels are typically doubled due to defective clearance of LDL particles by the liver. Individuals with untreated FH carries a substantial elevated risk of arteriosclerotic cardiovascular disease (CVD), especially coronary heart disease (CHD) [2].

Adherence to treatment is an important challenge in asymptomatic lifelong conditions in general [3], and as many as 30 % of young adults with FH have been shown to be non-adherent to their lipid lowering therapy (LLT) [4,5].

During 1980–1989, before statins were generally available, the Simone Broome Register Group reported a standardized mortality (SMR) for coronary heart disease of 3.86 in a cohort of 526 men and women with FH aged 20–74 years (SMR=1.00 for the normal population). The highest SMR ratio was nearly 97 in the age group 20–39 years, however with a very wide confidence Interval [6].

The introduction of hydroxy‑metylglutaryl-coenzyme A (HMG-CoA) reductase inhibitors, or statins, around 1990, revolutionized the treatment of FH with prospects for significant improvements in the prognosis of the disease. These hopes have only partly been confirmed in registry and cohort studies from many countries, including in the United Kingdom, Netherlands, Spain, Denmark and Norway [7]. Compared with the pre-statin era, the risk of CVD has been substantially reduced, but a considerable increased risk remains, even in treated FH, especially when treatment starts late in life and in secondary prevention [[8], [9], [10], [11]].

An impactful clinical experience is witnessing a young person with FH suffer a preventable heart attack, prompting us to study this issue among younger individuals with FH, during a period when effective treatment is readily available.

## 2 Aims

In a cohort of young individuals with genetically verified FH, to describe the risk of cardiovascular endpoints and all cause death during 2008–2018, compared to matched controls. In a subset of the same cohort, to describe use of LLT during 2004–2018. For statins and ezetimibe, also to describe proportion of days covered with dispensed prescriptions, proportion of patients covered with dispensed prescriptions, and time until first discontinuation of dispensed prescriptions.

## 3 Methods

This is a register-based study with linkage of the following Norwegian registers: The Unit for Cardiac and Cardiovascular Genetics (UCCG) Registry, with information on all individuals with a verified molecular genetic diagnosis of familial hypercholesterolemia from 1998; The Cause of Death Registry; The Norwegian Patient Registry (NPR,) containing information on all hospitalizations and specialist consultations with ICD-10 diagnoses from 2008 and onwards; The Cardiovascular Disease in Norway (CVDNOR) project, a research project including CV events in Norway during 1994–2009; The Norwegian prescription database (NorPD) including data on all dispensed prescriptions in all pharmacies in Norway from January 1st 2004 and onwards. Drugs administered during hospitalizations, clinical trials or stays within other institutions are not included. Data were linked across registries using the personal identification number, which is unique to each resident in Norway.

In the current paper we included individuals with a genetic diagnosis of heterozygous FH, born 1988–2008 (To reach a minimum of 10 years and a maximum of 30 years of age at end of follow-up in December 2018). Individuals were required to be alive January 1st 2008 (when follow-up for CVD-endpoints starts).

Patients were tested genetically from 1992 and onwards. In index cases, a pathogenic FH mutation was detected by Sanger-sequencing and Multiplex Ligation-dependent Probe Amplification (MLPA) analysis of the LDL-receptor gene, Sanger-sequencing of the proprotein convertase subtilisin/kexin type 9 (PCSK9) gene, and testing for the mutation R3500Q in the apolipoprotein B (APOB) gene. Non-index patients were only tested for the specific mutation in the family. Mutations were evaluated as pathogenic at the start of our study.

For analysis of CVD-endpoints, 20 matched controls per FH-person was included. Controls were selected at random from the general population registry, matched on sex and year of birth, and required to be alive on the date the individual with FH got the FH-diagnosis.

The study was approved by The Regional Committee of Medical and Health Research Ethics for South-Eastern Norway (reference 2011/1343 REK Sør-Øst B), and by the Norwegian Data Protection Officer at Oslo University Hospital. The implementation of the study complies with the Declaration of Helsinki. Consent was required to be included in the FH study cohort, whereas no consent was needed to be included in the control cohort. No additional consent was needed to be included in the present study (but individuals in the FH cohort had the opportunity to withdraw from the study until May 1, 2014).

### 3.1 Risk of CVD-endpoints

CVD diagnostic endpoints were identified in the Norwegian Patient Registry and the Cause of Death Registry. An endpoint was defined as either a hospitalization with the endpoints as primary or secondary diagnosis or a death with the endpoints as underlying cause of death. Treatment endpoints were defined as a hospitalization with percutaneous coronary intervention (PCI) or coronary artery bypass graft (CABG).

Endpoint-data were available from 2008 through 2018. ICD10-CVD diagnostic endpoint codes and NCSP treatment codes are listed in Supplementary Table 2.

Follow-up time for each endpoint was calculated as time from either date of FH-diagnosis or January 1st 2008 (whichever occurred last) until the endpoint, death from other causes or December 31st 2018, whichever occurred first. The start of follow-up for the controls were set to the same date as the person with FH in the matched set.

Difference in risk of CVD-endpoints between FH and controls were analyzed using Cox-regression when there were sufficient number of events.

### 3.2 Use of lipid-lowering therapy

Dispensed prescriptions of statins, including combination-drugs, resins, ezetimibe and PCSK9-inhibitors were obtained from NorPD for the period 2004–2018 according to Anatomical Therapeutic Chemical (ATC) Classifications (Supplementary Table 1).

Simple measures of “any use” during follow-up was described for the entire group with FH (*n* = 1351). Measures of statin compliance was estimated in a subset of the cohort; subjects diagnosed with FH during 2004–2014 and with at least one statin prescription during 2004–2017 (*n* = 833). The first prescription was required to be before 2018 in order to have at least one year of follow-up for subsequent prescriptions. Persons diagnosed with FH before 2004 (when the NorPD registry was established) was excluded in the analyses of medication in order to have complete follow-up of prescriptions from time of diagnosis. Similarly, measures of ezetimibe compliance was estimated for FH-persons diagnosed with FH during 2004–2014 and with at least one ezetimibe prescription during 2004–2017 (*n* = 205).

Compliance-analyses were done separately for statins and ezetimibe with combination drugs included. Persons were assumed to consume one tablet per day and the end-date for a prescription was defined as prescription-date + number of tablets.

We allowed for up to 90 days of stockpiling, i.e. if a person picks up 100 tablets and comes back to the pharmacy to pick up 100 new tablets after 50 days, we assume that he/she consumed the remaining 50 tablets before starting on the new batch of 100 tablets.

Follow-up time in compliance-analyses was started at the date of the first prescription. When defining discontinuation we allowed for a 180-day grace period, which means that the person was defined to have a discontinuation if he/she does not pick up at new prescription within 180 days after the end-date for the previous prescription. Date of discontinuation was set to end-date of prescription plus grace period. We also did sensitivity analyses with 90-days grace period and with follow-up from the second prescription instead of the first prescription. Based on the above definitions, each person’s follow-up time was divided into time-periods of continuous use and no use. Time to first discontinuation was reported using Kaplan-Meier survival failure curves with censoring at death or end of follow-up (December 31st 2018). We also used Kaplan-Meier survival curves to study time from first discontinuation of statins until restart among those who had a discontinuation.

Proportion of days covered (PDC) was defined as the number of days with continuous use divided by the total follow-up time. A proportion >80 % was defined as high compliance, 40–80 % was defined as medium compliance and <40 % was defined as low compliance.

Proportion of patients covered (PPC) was analyzed using the methods described by Rasmussen et al. to describe treatment persistence [12]. The PPC methods estimates the proportion of living patients who are covered by treatment on a given day after treatment initiation. The numerator includes patients that are currently covered by the latest prescription, and the denominator consists of all patients that are still alive. Persons who has previously discontinued is allowed back into the analysis set if they restart treatment. End of follow-up was set to December 31st 2018 or death, whichever occurred first.

## 4 Results

A total of 1351 subjects with FH (born between 1988 and 2008) and 27,021 controls were included in the study. After exclusion of 6 controls who died before January 1st 2008, 1351 persons with FH and 27,015 controls were included in the final study population. Mean age (SD) at genetic FH-diagnosis was 10.3 (5.5) years, ranging from 0 to 25 years. Mean age (SD) at start of follow-up was 12.3 (5.4) years for both subjects with FH and control subjects. Before start of follow-up (1st Jan 2008) there was one stroke and one case of atrial fibrillation in the FH population. In the control population there were 8 strokes and 4 heart failure events (Table 1).

**Table 1:** Description of study population.

		FH	Controls
n		1351	27,015
Sex n ( %)			
	Women	653 (48.3)	13,048 (48.3)
	Men	698 (51.7)	13,967 (51.7)
Age at start of follow-up*, mean (SD)		12.3 (5.4)	12.3 (5.4)
Age at end of follow-up, mean (SD)**		21.8 (5.7)	21.8 (5.7)
Age at FH-diagnosis, median (min-max)		10 (0–25)	
Age at FH-diagnosis, mean (SD)		10.3 (5.5)	
Year of FH-diagnosis, n ( %)			
	1992–2003	264 (19.5)	
	2004–2014	1087 (80.5)	
Cardiovascular disease before start of follow-up***, n			
	Previous CHD	0	0
	Previous Stroke	1	2
	Previous AF	1	0
	Previous HF	0	1
	Previous PAD	0	0
	Previous CABG/PCI	0	0
Use of lipid-lowering drugs during follow-up**** n ( %)			
	Statins	1126 (83.4)	0
	PCSK9-inhibitors	7 (0.5)	0
	Ezetimibe	281 (20.8)	0
	Resins	29 (2.2)	0

Abbreviations: CHD=Coronary Heart Disease. AF=Atrial fibrillation. HF=Heart Failure. PAD=Peripheral Artery Disease. CABG=Coronary Artery Bypass Graft. PCI=Percutaneous Coronary Intervention.

* January 1st 2008 for persons diagnosed with FH before 2008 and date of FH-diagnosis if diagnosed with FH after January 1st 2008.

** Calculated among those still alive at end of follow-up.

*** Checked both in NPR-data from 2008 until FH-diagnosis (for those with FH-diagnosis after 2008) and in CVDNOR-data 1994–2008.

**** At least one prescription during follow-up.

### 4.1 Cardiovascular disease and all-cause death

Incident acute myocardial infarction (AMI), was borderline increased in the FH-group, with one case (man 24 years old, not having dispensed prescriptions for LLT in the last 5 years before the infarction) and 2 cases in the control group (men 18 and 23 years old), HR (95 % CI) 10.0 (0.91–110.41), *p* = 0.06.

CHD was non-significantly increased in the FH-group, with the one case mentioned above and 3 cases in the control group, hazard ratio (HR) (95 % CI) 6.68 (0.69–64.20), *p* = 0.10.

All-cause death was borderline increased in the FH-group, with 6 cases (0.44 %) in the FH group and 53 cases (0.20 %) in the control group, HR 2.26 (0.98–5.28) *p* = 0.06. However, none of the deaths in the FH-group were related to cardiovascular disease.

Incidence of atrial fibrillation was not significantly different between groups and there were no strokes, heart failures or peripheral arterial disease (PAD) in the FH-population (Table 2).

**Table 2:** Cardiovascular endpoints and total mortality, 2008–2018.

	Events FH (*n* = 1351)	Events controls (*n* = 27,015)	HR (95 % CI)*	p-value
CHD**	1	3	6.68 (0.69–64.20)	0.10
AMI**	1	2	10.0 (0.91–110.41)	0.06
Stroke	0	14		
AF	1	11	1.82 (0.24–14.10)	0.57
HF	0	6		
PAD	0	6		
CABG/PCI**	1	0		
All-cause death	6	53	2.26 (0.98–5.28)	0.06

n for all variables.

Abbreviations: CHD=Coronary Heart Disease. AF=Atrial fibrillation. HF=Heart Failure. PAD=Peripheral Artery Disease. CABG=Coronary Artery Bypass Graft. PCI=Percutaneous Coronary Intervention.

* FH versus controls.

** Since AMI and CABG/PCI are parts of CHD, these events are also included in the CHD-group.

### 4.2 Use of lipid lowering drugs

In the entire FH-group (*n* = 1351), 83 % and 21 % of subjects had been prescribed a statin and/or ezetimibe respectively, at least once (during 2004–2018). Use of PCSK9-inhibitors and resins were diminutive. None in the control population had been prescribed lipid lowering drugs (Table 1).

### 4.3 Statins

Mean age (SD) at first statin prescription was 15.6 (4.0) and 16.2 (4.0) years for men and women respectively (*p* = 0.06). The proportion who started on statins before 15 years of age, was significantly higher among men than among women (44.1 % versus 36.5 %, *p* = 0.03). In the group investigated for compliance with LLT (*n* = 833) mean (SD) PDC was 0.84 (0.22), ranging from 0.34 to 1 (5th-95th percentile). Approximately 69 % of subjects had >80 % of days covered and approximately 7 % had <40 % of days covered. In those aged 15 years or older, compared to those below 15 years at diagnosis, compliance was somewhat lower (Table 3).

**Table 3:** Use of lipid lowering therapy and compliance of statin-use measured as percent of days covered (PDC) among persons diagnosed with FH during 2004–2014 and with at least one statin-prescription during 2004–2017, followed until the end of 2018.

		Total	Men	Women	p-value**
n ( %)		833	422 (50.7)	411 (49.3)	
Age at FH-diagnosis, years, mean (SD)		11.6 (5.5)	11.3 (5.5)	11.9 (5.5)	0.08
Age at first statin-prescription, years, mean (SD)		15.9 (4.0)	15.6 (4.0)	16.2 (4.0)	0.06
Age-group at first statin-prescription, n ( %)					
	<15 years	336 (40.3)	186 (44.1)	150 (36.5)	0.03
	≥15 years	497 (59.7)	236 (55.9)	261 (63.5)	
N statin-prescriptions, median (p5-p95)		13 (2–33)	13 (2–34)	13 (3–33)0.66	
Only one statin-prescription, n ( %)		15 (1.8)	8 (1.9)	7 (1.7)	0.83
Any use of ezetimibe, n ( %)		238 (28.6)	111 (26.3)	127 (30.9)	0.14
Any use of resins, n ( %)		16 (1.9)	5 (1.2)	11 (2.7)	0.12
Any use of PCSK9-inhibitors, n ( %)		4 (0.5)	2 (0.5)	2 (0.5)	0.98
PDC*, mean (SD)		0.84 (0.22)	0.84 (0.23)	0.83 (0.22)	0.61
PDC*, median (p5-p95)		0.96 (0.34–1)	0.97 (0.33–1)	0.94 (0.36–1)	0.03
PDC in categories n ( %)					
	< 0.40	58 (7.0)	31 (7.4)	27 (12.9)	0.13
	0.40–0.80	198 (23.8)	88 (20.9)	110 (26.8)	
	> 0.80	577 (69.3)	303 (71.8)	274 (66.7)	
PDC, age <15 years at first statin prescription					
	< 0.40	6 (1.8)	3 (1.6)	3 (2.0)	0.69
	0.40–0.80	63 (18.8)	32 (17.2)	31 (20.7)	
	> 0.80	267 (79.5)	151 (81.2)	116 (77.3)	
PDC, age ≥15 years at first statin prescription					
	< 0.40	52 (10.5)	28 (11.9)	24 (9.2)	0.21
	0.40–0.80	135 (27.2)	56 (23.7)	79 (30.3)	
	> 0.80	310 (62.4)	152 (64.4)	158 (60.5)	

Abbreviations: PDC=Proportion of days covered.

*A grace-period of 180 days has been used to define periods of continuous use.

** P-values calculated using *t*-test for comparison of means, median test for comparison of medians and chi-square test for comparison of proportions.

Chi-square test for association between age group and categorical PDC: *p* < 0.001.

**Fig. 1:**
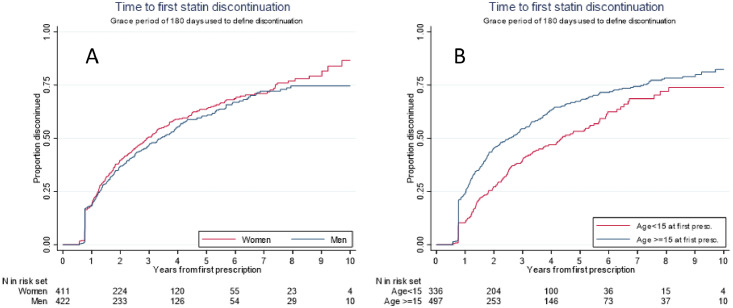
Drug survival for statin-use among *n* = 833 FH-persons diagnosed with FH during 2004–2014 and with at least one statin-prescription during 2004–2017. Follow-up time from first statin-prescription to first discontinuation. Grace-period of 180 days used for definition of discontinuation.Panel A: Men and women. Panel B: Age<15 years and Age≥ 15 years.

**Fig. 2:**
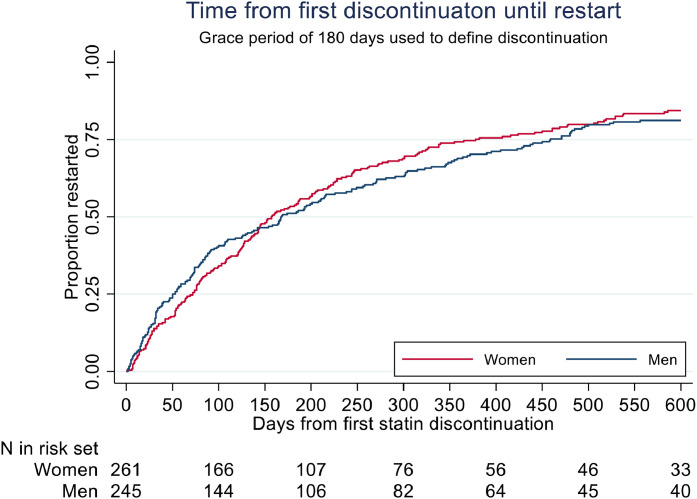
Time to restart on statins after first discontinuation among 506 persons with FH where the first period of statin use ended with a discontinuation.

With at grace period of 180 days (i.e. do not pick up a new prescription within 180 days after the end-date for the previous prescription), 18.5 % of the subjects have their first discontinuation of statins within 1 year after the first prescription, with more discontinuations among those aged 15 years or older at first prescription (Fig. 1). Among those who discontinue (506 out of 833=60.7 %), 34 % of women and 41 % of men have restarted statin therapy within 100 days after the first discontinuation (Fig. 2).

**Fig. 3:**
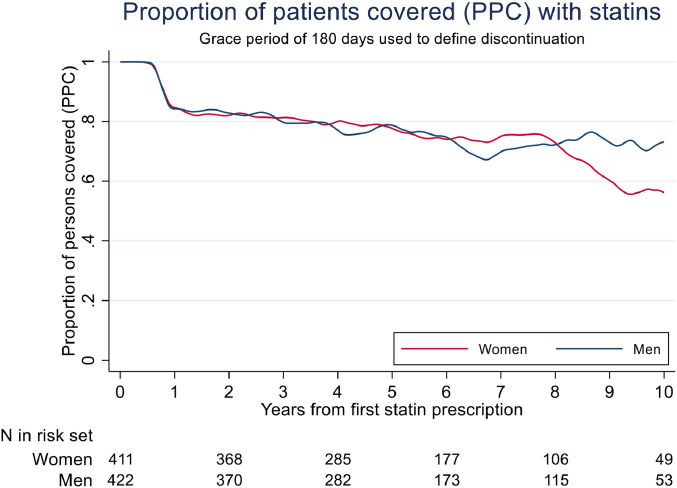
Proportion of patients covered (PPC) for statin-use among *n* = 833 FH-persons diagnosed with FH during 2004–2014 and with at least one statin-prescription during 2004–2017. Grace period of 180 days used to define discontinuation. Curves are smoothed using lowess running mean smoothing.

Conversely, looking at proportion of patients covered (PPC) with statins over time, with restarters allowed back into the analyses, the proportion covered drops during the first year, with less reduction thereafter. One year after initiation of statins PPC is approximately 85 %, and at 8 years approximately 70 % in both men and women, thereafter statin use is stable in men but with a drop in statin use among women (Fig. 3).

### 4.4 Ezetimibe

Mean age at first ezetimibe prescription was 20.7 years, with no significant differences between men and women. Compliance of ezetimibe-use is similar to statins with 60 % of subjects having >80 % of days covered and 19 % having <40 % of days covered.

**Fig. 4:**
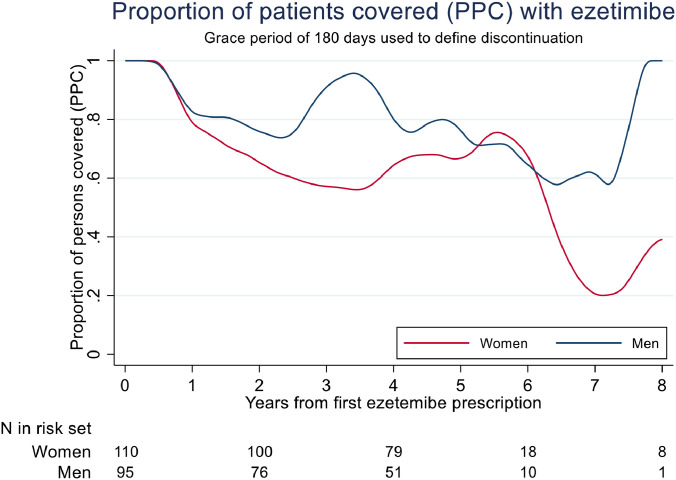
Proportion of patients covered (PPC) for ezetemibe-use among *n* = 205 FH-persons diagnosed with FH during 2004–2014 and with at least one ezetemibe-prescription during 2004–2017. Grace period of 180 days used to define discontinuation. Curves are smoothed using lowess running mean smoothing.

As with statins, approximately 25 % of subjects who discontinue ezetimibe do so within 1 year after the first prescription. Thereafter more women than men discontinues ezetimibe. As for statins, the PPC drops substantially during the first year, with less reduction thereafter, but more so in women than in men, and with larger fluctuations over time than for statins. (Fig. 4 and supplementary Figs. 5–7).

### 4.5 Sensitivity analyses

When using grace-period of 90 days, time to first discontinuation of statins and ezetimibe shows similar patterns as with a 180 days grace period, with a distinct step in discontinuations during the first year, appearing earlier with 90 days grace period compared to 180 days grace-period. Also, analyses with follow-up from the second prescription instead of the first prescription showed a similar pattern of discontinuations. (Supplementary Fig. 2).

## 5 Discussion

While increased cardiovascular risk in the adult population with FH, also in recent years, have been well documented [7], data on long-term cardiovascular risk among young individuals with FH are very sparse.

To our knowledge our study is the largest cohort of young individuals with FH, monitored long-term for cardiovascular disease and all-cause death. In our group of 1351 subjects, all with a verified pathogenic FH-mutation, aged up to and including 30 years, incidences of CVD and all-cause deaths seems to be low after 11 years of follow-up. There was only one CHD event, and none of the 6 deaths among the subjects with FH were related to CVD. Although our results may suggest increased risk of CHD and all-cause death relative to the general population, measures are imprecise due to the low number of events and low absolute risks. The one CV event in our cohort with FH was in a non-compliant 24-year-old male, untreated for the last 5 years. In Norway, we can assume that all severe ASCVD events in young individuals will lead to hospital or specialist contact. With complete registration, we have thus documented a very low incidence of CV events. Although our results are not statistically significant, this is reassuring for clinicians and patients. Our results are in line with the results from a twenty-year follow-up study of approximately 200 subjects with FH in The Netherlands, treated with statins from 13 years of age [13]. A CV event (angina pectoris) occurred in one individual (1 %), as compared with 41 out of 156 (26 %) in their parents with FH, up to 40 years of age. CV death occurred in 0 % vs. 7 %, in children and parents respectively, underscoring the importance of early diagnosis and initiation of treatment in FH. In The Netherlands-study, however, no comparison with the general population was done.

In a somewhat older Norwegian cohort of 2795 adults with FH followed during 2001–2009 [14], the highest relative risk for CHD was among subjects aged 25–39 years at start of follow-up, with significantly increased standardized incidence ratios of 11.1 in men and 17.3 in women (as compared to the general population), which is within the confidence interval for estimated risk of CHD in our cohort. In the study with the older cohort, mean age at genetic diagnosis, however, was almost 32 years as compared to 10 years in our cohort, implying later initiation of statin therapy.

Compliance with statins in our study is fairly good with a little less than 85 % of individuals covered after one year, slowly decreasing over the years to approximately 70 % covered after 8 years. The drop in coverage among women after 8 years is probably due to pregnancies and breastfeeding. Previously we have shown that women loose in average 2.3 years of statin treatment at the age of 31 years, constituting approximately 20 % of their potential treatment time [15]. Previously we have shown that 30 % of children and young adults treated with statins have adherence issues, with higher age, more visits and more years of follow-up associated with good adherence [4]. Still, boys are initiated on statin therapy at a younger age than girls, despite the fact that adolescent girls have higher LDL-C levels than boys [16]. We do not know why, other than it may be a treatment practice that lingers from the past, when treatment advice was not to initiate statins in girls until one year after menarche.

Discontinuations of therapy shows a pattern with a sharp increase during the first year after initiation of therapy and a slower rise in discontinuations thereafter. The sharp increase in discontinuation is caused by a rather large proportion having a long gap between the first and second prescription, causing all of them to be defined as discontinuers after approximately 280 days (100 tablets in first prescription + 180 days grace period). In addition, there are some with only one prescription, which will also be defined as discontinuers after 280 days. This pattern persists also when starting follow-up at the second prescription, indicating that these patients have longer time periods between prescriptions in general, probably because of sporadic use or treatment pauses.

In clinical practice it is a common observation that many have shorter or longer pauses in the drug treatment, especially among young adults who feel healthy (and even sometimes immortal), and who do not fully understand the long-term risk of being untreated [17]. Our study confirms this observation; a majority of patients discontinue treatment at some time during follow-up, but reassuringly, most of them restarts therapy within 6 months. The greatest cause for worry, however, is for the 7 % of the subjects with less than 40 % of days covered with statins. When LDL-C levels are high, as in FH, longer untreated periods will add substantially to the lifelong cumulative LDL-C load. Visit to visit LDL-C variability has also been shown to be a predictor of CV events in subjects with coronary artery disease [18].

After the first statin prescription, the routine in our clinic is that the patient make an appointment with the general practitioner for blood tests after 2–3 months, and a new appointment in the clinic after one year. Practice may vary between lipid clinics, but in view of these results, a lipid clinic consultation 6 months after the first prescription should be considered to ensure adherence.

Results for compliance with ezetimibe are principally similar to the results for statins. However, ezetimibe treatment is initiated 5 years later than statins (mean 21 years and 16 years of age, respectively). This is probably the explanation for the sharp drop among women in proportion of patients covered within the first year after initiation of therapy, since a large proportion of the women are in an age group where it is common to plan pregnancies or become pregnant.

Increasing age is a strong risk factor for CV and the low incidences of CVD in our cohort may not be surprising, even with a low drug compliance among some individuals. Smoking, a strong risk factor for CVD in combination with hypercholesterolemia, has also declined sharply in recent years. In 2023 only 3 % of those aged 16–24 years in the general population in Norway were daily smokers, a reduction from 7 % during the last 10 years [19]. Among those with FH smoking is probably even less common[20], possibly contributing to the low event rates.

## 6 Strengths and limitations

Strengths are the high number of subjects, all with genetically verified FH and the nationwide registers with unique individual identification, ensuring a complete follow up. Even so, due to the low incidence rates for CVD, the number of subjects with FH are too low for statistical power to demonstrate differences in CVD as compared to the general population. The absence of serum lipid levels in both the FH and the control cohort, as well as lack of information about smoking and other cardiovascular risk factors, is a limitation

During 2010–2013, 60 children from our clinic, aged 6–17 years participated in two open-label clinical trials and were treated with rosuvastatin or atorvastatin [21,22]. Study drugs were supplied from the sponsors and not registered in the NorPD, possibly biasing our results towards lower compliance. Also, in some families several persons may use the same drugs and may irregularly use from each other’s prescriptions, potentially biasing towards lower drug compliance.

## 7 Conclusion

Risk of cardiovascular disease in young subjects with FH is difficult to assess due to low incidence rates. Long term compliance to LLT is fairly good in the entire group, but challenging for some. It may be beneficial for compliance to initiate statin therapy before 15 years of age. We still have a way to go in motivating some of our young patients to take their statins, which is the simplest method to prevent early CVD.

## Author agreement

All authors agree to be accountable for all aspects of the work

## Funding

South-Eastern Norway Regional Health Authority and Throne Holst Foundation.

## Data availability

With permission from the regional ethical committees and the appropriate national authorities and register holders, data from the registers utilized in the study are accessible at: https://helsedata.no/en/. The specific data files utilized for this investigation are not publicly accessible due to data privacy regulations.

## CRediT authorship contribution statement

**Gisle Langslet:** Writing – original draft, Methodology, Conceptualization. **Emil A. Asprusten:** Writing – review & editing, Conceptualization. **Jannicke Igland:** Writing – original draft, Software, Methodology, Formal analysis, Data curation, Conceptualization. **Kirsten B. Holven:** Writing – review & editing. **Martin P. Bogsrud:** Writing – review & editing. **Kjetil Retterstøl:** Writing – review & editing, Supervision, Project administration, Methodology, Funding acquisition, Conceptualization.

## Declaration of competing interest

Disclaimers: GL received honoraria/coverage of expenses from Ultragenyx. EAA received honoraria from Amarin, Novartis and Sanofi. JI received funding from Sanofi and Novartis to conduct postmarketing drug safety research. KBH received honoraria from Sanofi. MPB received honoraria/coverage of expenses from Amgen, Novartis, Sanofi and Ultragenyx. KR received honoraria/coverage of expenses from Amgen, Amarin, Novartis, Novo Nordic and Sanofi. All declarations are outside the submitted work.

## Appendix Supplementary materials

Image, application 1
