# Supplementary material for: Cardiovascular disease and compliance with lipid-lowering therapy among young individuals with familial hypercholesterolemia in Norway – A register study
Source: Am J Prev Cardiol. 2025 Jun 18;23:101043. doi: 10.1016/j.ajpc.2025.101043 (PMC12242432; doi:10.1016/j.ajpc.2025.101043)
Supplement: Supplementary file 1 [file mmc1.docx]

Supplementary Material

Supplementary tables:

*Supplementary table 1: ATC-codes*

| Drug group | ATC-codes |
| --- | --- |
| Statins, including combination-drugs | C10AA, C10BA01, C10BA05, C10BA06 |
| Resins | C10AC |
| Ezetimibe | C10AX09, C10BA01, C10BA05, C10BA06 |
| PCSK9-inhibitors | C10AX13, C10AX14 |

*Supplementary table 2: CVD diagnostic ICD10-codes and CVD treatment NCSP-codes*

| Endpoint | ICD10-codes | NCSP-codes |
| --- | --- | --- |
| Coronary Heart Disease | I20-I25 |  |
| Acute Myocardial Infarction | I21-I22 |  |
| Total stroke | I61, I62,I63 (excl. I63.6),I64 |  |
| Peripheral artery disease | I70, I73 |  |
| Atrial fibrillation and flutter | I48 |  |
| Heart failure | I50 |  |
| PCI/CABG |  | FNG02, FNG05, FNOB00, FNO10. FNP02, FNP12, FNQ05, FNQ12, FNA, FNB, FNC, FND, FNE |
| All-cause death |  |  |

Supplementary table 3: Compliance of ezetimibe-use measured as percent of days covered (PDC) among persons diagnosed with FH during 2004-2014 and with at least one ezetimibe-prescription during 2004-2017, followed until the end of 2018.

|  | | Total | Men | Women | p-value** |
| --- | --- | --- | --- | --- | --- |
| n | | 205 | 95 | 110 |  |
| Age at FH-diagnosis, mean (SD) | | 13.3 (5.0) | 12.5 (5.1) | 14.1 (4.9) | 0.02 |
| Age at first ezetimibe-prescription, mean (SD) | | 20.7 (4.1) | 20.2 (4.4) | 21.2 (3.7) | 0.09 |
| Age-group at first ezetimibe -prescription, n (%) | |  |  |  |  |
|  | <15 | 12 (5.9) | 10 (10.5) | 2 (1.6) | 0.01 |
|  | ≥15 | 193 (94.2) | 85 (89.5) | 108 (98.2) |  |
| N ezetimibe -prescriptions, median (p5-p95) | | 6 (1-21) | 6 (1-22) | 6 (1-20) | 0.99 |
| Only one ezetimibe -prescription, n (%) | | 16 (7.8) | 7 (7.4) | 9 (8.2) | 0.83 |
| Any use of statins, n (%) | | 205 (100) | 95 (100) | 110 (100) |  |
| Any use of resins, n (%) | | 10 (4.9) | 2 (2.1) | 8 (7.3) | 0.09 |
| Any use of PCSK9-inhibitors, n (%) | | 3 (1.5) | 2 (2.1) | 1 (0.9) | 0.48 |
| PDC*, mean (SD) | | 0.83 (0.25) | 0.88 (0.22) | 0.78 (0.27) | 0.005 |
| PDC*, median (p5-p95) | | 0.99 (0.29-1) | 1 (0.29-1) | 0.92 (0.24-1) | 0.006 |
| PDC in categories | |  |  |  |  |
|  | < 0.40 | 22 (10.7) | 7 (7.4) | 15(13.6) | 0.02 |
|  | 0.40-0.80 | 43 (21.0) | 14 (14.7) | 29 (26.4) |  |
|  | > 0.80 | 140 (68.3) | 74 (77.9) | 66 (60.0) |  |

* PDC=Proportion of days covered. A grace-period of 180 days has been used to define periods of continuous use.

**P-values calculated using t-test for comparison of means, median test for comparison of medians and chi-square test for comparison of proportions.

Supplementary figures:

Supplementary Figure 1: Drug survival for statin-use among n=873 FH-persons diagnosed with FH during 2004-2014 and with at least one statin-prescription during 2004-2017. Follow-up time from first statin-prescription to first discontinuation. Grace-period of 90 days used for definition of discontinuation.

.

Supplementary Figure 2. Drug survival for statin-use among n=818 FH-persons diagnosed with FH during 2004-2014 and with **at least two** statin-prescriptions during 2004-2017. Follow-up time from **second** statin-prescription to first discontinuation. Grace-period of 180 days used for definition of discontinuation.

Supplementary Figure3: Proportion of patients covered (PPC) for statin-use among n=833 FH-persons diagnosed with FH during 2004-2014 and with at least one statin-prescription during 2004-2017. Grace period of 90 days used to define discontinuation. Curves are smoothed using lowess running mean smoothing.

Supplementary Figure 4: Proportion of patients covered (PPC) for statin-use among n=833 FH-persons diagnosed with FH during 2004-2014 and with at least one statin-prescription during 2004-2017, stratified by age at first statin prescription. Grace period of 90 days used to define discontinuation. Curves are smoothed using lowess running mean smoothing.

Supplementary figure 5: Drug survival for ezetimibe-use among n=205 FH-persons diagnosed with FH during 2004-2014 and with at least one ezetimibe-prescription during 2004-2017. Follow-up time from first statin-prescription to first discontinuation. Grace-period of 180 days used for definition of discontinuation.

Supplementary Figure 6: Drug survival for ezetimibe-use among n=205 FH-persons diagnosed with FH during 2004-2014 and with at least one ezetimibe-prescription during 2004-2017. Follow-up time from first statin-prescription to first discontinuation. Grace-period of 90 days used for definition of discontinuation.

Supplementary Figure 7: Proportion of patients covered (PPC) for ezetimibe-use among n=205 FH-persons diagnosed with FH during 2004-2014 and with at least one ezetimibe-prescription during 2004-2017. Grace period of 90 days used to define discontinuation. Curves are smoothed using lowess running mean smoothing.
